# Effects of weight loss on foot structure in obese adults: a pilot study

**DOI:** 10.1186/1757-1146-5-S1-O48

**Published:** 2012-04-10

**Authors:** Jinsup Song, Gary Foster, Reagan Kane, Dana N Tango, Stephanie Vander Veur, Naomi Reyes, Caitlin LaGrotte, James Furmato, Eugene Komaroff

**Affiliations:** 1 Gait Study Center, Temple University, Philadelphia, PA, USA; 2 Center for Obesity Research and Education, Temple University, Philadelphia, PA, USA

## Background

Excessive body weight can have a profound influence on weight bearing structure and function, including pain, disability, and compromised quality of life. A prospective cohort study of 5,784 people over the age of 50 years, showed that obesity was a strong predictor of the onset of severe disabling knee pain.[1] Similarly, increased weight was found to have an association with chronic plantar heel pain syndrome.[2] No study to-date has conducted an objective prospective examination of the differences in foot structure and function during significant weight change.

## Materials and methods

In this randomized controlled prospective pilot study, 41 obese subjects were randomly assigned to either the treatment or the control group. Subjects assigned to the treatment group received weekly education plus pre-packaged portion-controlled meals while the control group received monthly education only. Foot structure measurements (malleolar valgus index and arch height) were assessed at baseline and 3-month. Repeated ANOVA (Matched Pairs by Group) analysis was performance using JMP 9.

## Results

The mean age of study participants was 56.2 years old. While there was no difference in body mass index between the two groups at baseline, the treatment group lost significant weight at 3-month, see Table 1.

**Table 1:** Summary of foot structure measures of the control and the treatment groups at baseline and 3-month

	Control		Treatment		p-value
		
	Baseline	3-month	Baseline	3-month	
Body Mass Index ^a^	35.8	35.1	36.1	34.0	<0.0001
Malleolar Valgus Index (%)	12.9	12.9	13.4	12.7	0.3103
Standing arch height (cm)	6.11	6.18	5.99	6.02	0.5593
Arch drop (cm)	0.44	0.40	0.43	0.47	0.0828

^a^ Control group lost an average of 1.9 kg (2.9%) of body weight while the treatment group lost 5.9 kg (6.2%) at 3-month visit.

**Figure 1:**
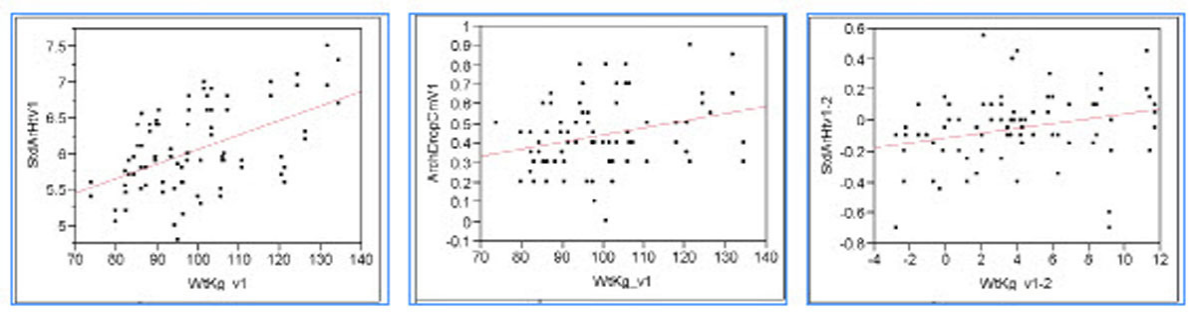
Linear correlation of (a) body weight and standing arch height at baseline [StdArchHt = 4.036 + 0.020 * Weight, p<0.0001], (b) body weight and arch drop at baseline [Arch drop= 0.075 + 0.003 * weight, P=0.006], and (c) change in weight and change in standing arch height [ ∆StdArchHt = -.0114 + 0.016 * ∆ Weight, P=0.013]

## Conclusions

No significant changes were noted in measured structural foot parameters at 3-month follow up between the two groups as a function of observed weight loss. It is not clear if a larger weight reduction would have yielded significant changes. Several foot dimensions (including standing arch height and arch height drop from the sitting to standing conditions) were linearly correlated with body weight.
